# A Comparative Study of Ultrasound-Guided Quadratus Lumborum Block and Transversus Abdominis Plane Block for Postoperative Analgesia Following Total Abdominal Hysterectomy

**DOI:** 10.7759/cureus.36412

**Published:** 2023-03-20

**Authors:** Usha Shukla, Urvashi Yadav, Jasleen Duggal

**Affiliations:** 1 Anaesthesiology, Uttar Pradesh University of Medical Sciences, Etawah, IND

**Keywords:** nrs, ultrasound, quadratus lumborum block, transversus abdominis plane block, postoperative analgesia

## Abstract

Background: Total abdominal hysterectomy (TAH) results in remarkable postoperative pain. Truncal nerve blocks like the quadratus lumborum (QL) block and transversus abdominis plane (TAP) block are described to combat this pain. The aim of the present study was to compare the effectiveness of the QL block with the TAP block in terms of numeric rating scale (NRS) scores as the primary outcome for postoperative pain in TAH. The secondary outcome was time of the first analgesic demand, total analgesic demands required in 24 hr, and patient satisfaction level.

Methods: Seventy patients of American Society of Anesthesiologists (ASA) physical status class I and II, aged 35-65 years with a body mass index (BMI) 18-30 kg/m^2^ planned for elective TAH under spinal anaesthesia were randomly allocated into two groups. Group Q received QL block and Group T received TAP block under ultrasound guidance with 40 ml of 0.25% bupivacaine of which 20 ml was injected on either side.

Results: Demographic data of both groups was comparable. The NRS pain score was significantly lower in Group Q compared to Group T at the fourth^ ^and sixth^ ^hour. The mean first analgesic demand was significantly early in Group T compared to Group Q (5.69 ± 0.87 hr vs. 11.23 ± 2.22 hr) and total analgesic demands were significantly greater in Group T than Group Q. The mean patient satisfaction score was significantly high in Group Q compared to Group T (5.8 ± 0.41 vs. 4.74 ± 0.44).

Conclusion: The combined posterior and anterior approach of the QL block may represent a more efficacious alternative to the TAP block in patients after TAH. Further studies are recommended to evaluate the ideal dose, volume and approach for the QL block.

## Introduction

Abdominal hysterectomy is the most common surgery performed for various malignant and benign conditions like uterine leiomyoma, cervical or uterine cancers or pelvic organ prolapse [1]. Open abdominal hysterectomy is a major surgery that leads to severe postoperative pain; ineffective pain relief hinders smooth postoperative recovery [2-5]. Acute postoperative pain causes late recovery, increases hospital stay, immediate infectious, neurological, cardiovascular, thromboembolic complications and long-term consequences like post-hysterectomy or hysterectomy chronic pelvic pain syndrome [6]. Analgesic options for effective pain management in intra-abdominal surgeries include systemic analgesia, regional analgesia, local infiltration, and abdominal nerve blocks. Anterior abdominal wall nerve blocks such as the transversus abdominis plane (TAP) block and quadratus lumborum (QL) block are effective for postoperative analgesia. Ultrasound guidance allows real-time visualization of nerves, surrounding structures, and the needle-tip to maximize block success and minimize complications [7]. The TAP block provides sensory block of thoracolumbar nerves (T6 to L1). Blocking these nerves provides analgesia to the anterolateral abdominal wall [8]. Limitations of the TAP block include a short duration of action and providing only somatic pain relief.

The QL block provides extended sensory blockade and is currently used as an effective analgesic tool for all age groups (pediatrics, pregnant, and adult) undergoing abdominal surgery. Currently, four approaches are described: lateral or type 1, anterior or type 2, posterior or type 3, and intramuscular or type 4. The spread of local anesthetic varies with each approach. The dermatomal coverage described for lateral and posterior approaches is from T7 to L1, for anterior approach from T10 to L4 and for intramuscular approach is T7 to T12. However, the best approach is yet to be determined. We studied the combined anterior and posterior approach to increase the dermatomal spread as it involves injecting the local anesthetic more posteriorly compared to the TAP block, therefore benefitting from cephalad spread to thoracic paravertebral space [9]. The inhibition of sympathetic fibers in the space is responsible for extensive, long-lasting effect and the potential to provide somatic as well as the visceral analgesia. Therefore, we planned this study to compare the effectiveness of QL block given by the combined posterior and anterior approach and TAP block for postoperative pain following TAH.

## Materials and methods

After receiving approval from the Institutional Ethical Committee of Uttar Pradesh Medical University of Medical Sciences (EC no. 152/2020-21) and the patients' informed consent, this prospective, randomized double-blind comparative trial was conducted over a period of one year. The formula used to get the sample size was n=4pq/L^2^, where n is the sample size, p is the approximate prevalence for which the trial is to be done (found in a prior study by Sen et al.), q = 1-p, and L is the permissible error in the estimate; it yielded a result of 70 with 80% power [5].

A total of 70 females of American Society of Anesthesiologists (ASA) physical status class I and II, aged 35-60 years, with BMI 18-30 kg/m^2^, planned for elective open total abdominal hysterectomy (TAH) under spinal anaesthesia were included in the study. Patients with a neurological disorder, renal or hepatic impairment, cardio-respiratory dysfunction, coagulopathy, localized infection at the injection site, allergy to the study drugs and opioid tolerance/dependence were excluded.

A computer-generated randomization table was used to assign patients at random to two groups. The block administered was not known to the patients and the observer who recorded all pain scores. All patients were given instructions on how to use the numeric rating scale (NRS), which ranged from 0 (no pain) to 10 (the most intense pain they had ever experienced), and were asked to choose the number that most accurately described their level of discomfort. Patients were required to fast for eight hours before the scheduled surgery time. The day before surgery, all patients were given 150 mg of ranitidine and 0.25 mg of alprazolam by mouth at night. Baseline hemodynamic parameters were recorded along with standard monitoring, which included noninvasive blood pressure (NIBP), heart rate (HR), oxygen saturation (SpO_2_), and electrocardiography (ECG). All patients received an intravenous (IV) cannula of the appropriate size, and a 10-15 ml/kg Ringer's lactate infusion was started. Routine monitoring of HR, NIBP and SpO_2_ was done throughout the intra-operative and postoperative period.

TAH was carried out under the subarachnoid block using a 25G Quincke spinal needle and 3-3.5 ml of 0.5% bupivacaine HCL was administered in the L4/L5 intervertebral space. Following surgery, the sensory level was assessed using the cold spray technique, and the proposed block (QL or TAP) was administered according to randomization using a portable ultrasound machine (Sonosite M-Turbo, with a curved transducer of 5-2 MHz; Fujifilm Medical Systems, Lexington).

In Group Q, QL block was carried out while the patient was positioned laterally. The quadratus lumborum, psoas major, and erector spinae muscles that make up the "shamrock sign" were visible when the ultrasound transducer was positioned transversely on the flank at the horizontal level of L2-3. The needle was inserted in the anterolateral to posteromedial direction. We performed combined type 2 and type 3 QL block bilaterally in Group Q. For the type 2 QL block, a local anesthetic was injected between the posterior surface of the QL muscle and thoracolumbar fascia. The point of injection in the type 3 QL block was between the anterior border of the QL muscle and psoas major muscles. A similar process was repeated on the contralateral side.

In Group T, with the patient in supine position, a transversus abdominis plane block was carried out. Between the lower costal border and the iliac crest, an ultrasound probe was positioned in a transverse plane. The 22G needle was inserted in the anterior axillary line after the transversus abdominis muscle was located beneath the external and internal obliques. The needle tip was then advanced until it reached the fascial plane between the internal oblique and transversus abdominis muscles, which was roughly in the midaxillary line, and 20 ml of 0.25% bupivacaine was deposited bilaterally. Real-time visualization of the local anesthetic solution's distribution was performed.

Postoperative pain was assessed by an independent observer blinded to the study using NRS, first at zero hour, i.e., immediately after the block, and then every two hours up to 12 hr and every four hours till 24 hr after the block. The duration of analgesia was considered from the time the study drug was placed to the time for the first demand of rescue analgesia. Whenever NRS was ≥4, intramuscular diclofenac (1.5 mg/kg) was administered (maximum 150 mg), and if it was not sufficient, then tramadol 1 mg/kg IV (maximum 200 mg) was added in 24 hr. Pain score in 24 hr, duration of analgesia and total analgesic demands were recorded. The patient's level of satisfaction was evaluated using a 7-point Likert verbal rating scale, where 1 denoted extreme dissatisfaction, 2 dissatisfaction, 3 slight dissatisfaction, 4 undecided, 5 slight satisfaction, 6 satisfaction and 7 extreme satisfaction [10]. During or after the procedure, adverse events such as bradycardia (HR 60 bpm or 20% decrease from the baseline value), hypotension (20% drop in the blood pressure from the baseline or an absolute mean arterial pressure, or MAP, 60 mm Hg), bradypnea (RR 8 breaths/min), desaturation (94%), nausea, vomiting, dry mouth, or any other events were noted.

The statistical analysis was performed using Minitab software (National Institute of Standards and Technology, Pennsylvania State University, PA). The quantitative variables were expressed as means ± SDs and were compared using the two-sample t-test. Categorical data was compared using the two-sample proportion test. A P value < 0.05 was considered statistically significant and a P value < 0.001 was considered highly significant.

## Results

**Figure 1:**
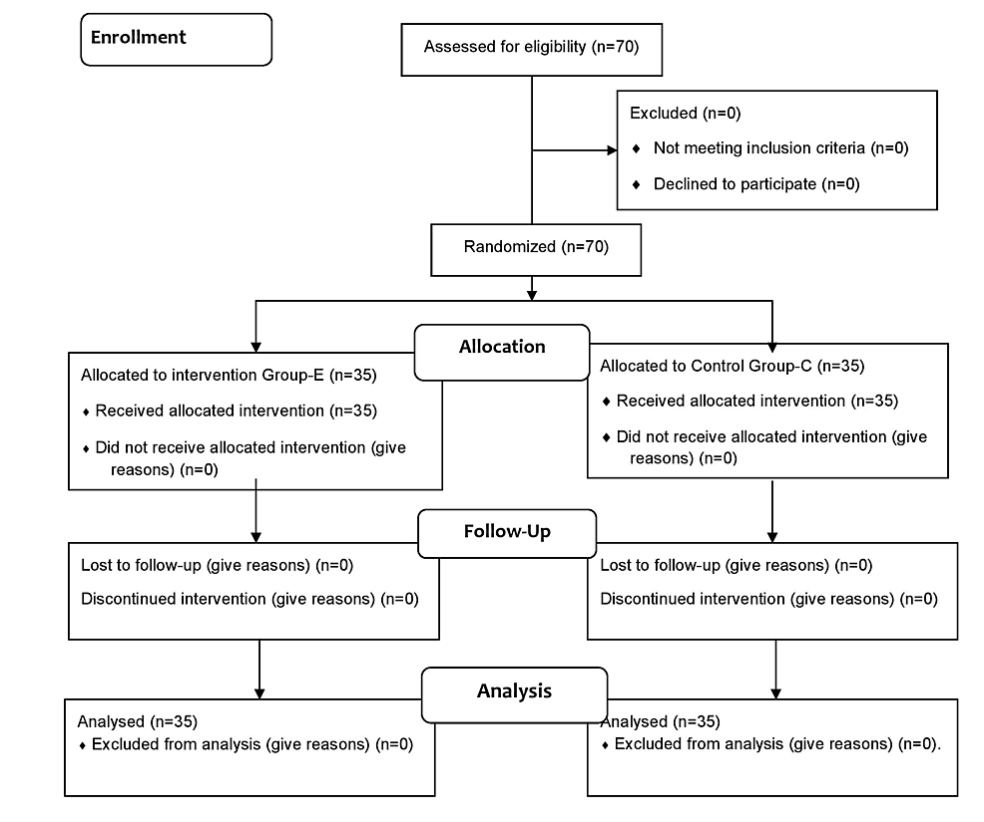
Consort flow diagram

All enrolled participants completed the study and none were excluded till the end of the study (Figure 1).

The groups were comparable with respect to demographic variables. The mean age of patients of Group Q was 46.69 ± 8.23 years and that for Group T was 46.37 ± 7.82 years. The mean BMI in Group Q was 24.31 ± 2.11 and in Group T was 23.94 ± 2.27. The mean duration of surgery in Group Q was 104.71 ± 21.11 min and in Group T was 98.43 ± 18.1 min. The percentage of patients in Group Q with ASA physical status class I was 60% and with class II was 40%; in Group T, the percentage with ASA class I was 65.7% and with class II was 34.3%. There was no significant difference in age, BMI, duration of surgery and ASA class between the groups on statistical analysis, showing the comparability between the groups (P > 0.05) (Table 1).

**Table 1:** Demographic data of the patients ASA = American Society of Anesthesiologists, BMI = body mass index, SD = standard deviation

		Group Q (n=35, mean ± SD)	Group T (n=35, mean ± SD)	P value
Age (years)		46.69 ± 8.23	46.37 ± 7.82	0.435
BMI (kg/m^2^)		24.31 ±2.11	23.94 ± 2.27	0.241
Duration of surgery (min)		104.71 ± 21.11	98.43 ± 18.1	0.093
ASA class	I	60%	65.7%	0.771
	II	40%	34.3%	

**Figure 2:**
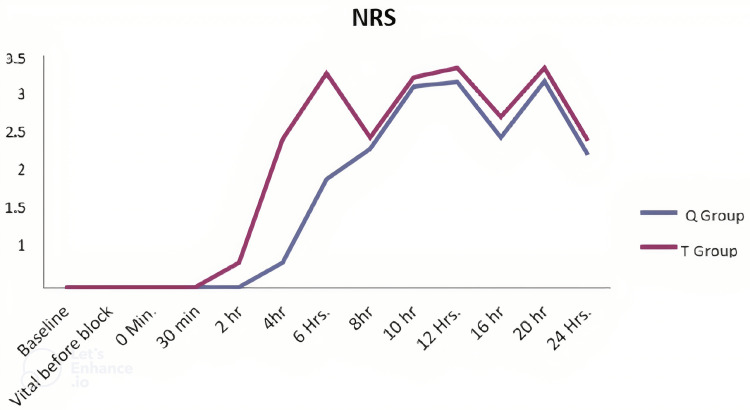
Comparison of NRS scores between the groups across the time periods NRS = numeric rating scale, Q = quadratus lumborum, T = transversus abdominis plane

At the time of the application of block, patients did not have any pain due to the effect of the subarachnoid block given for surgery. After 30 min of the application of block, patients started experiencing pain and the NRS score started to increase in Group T whereas the NRS score started to increase in Group Q after two hours but not as high as in Group T. The NRS score was significantly high in Group T, at the fourth hour (P < 0.001) and sixth hour (P < 0.001). After six hours, the NRS score of Group T decreased due to the effect of rescue analgesics used. The NRS score was less in the QL group than the TAP group at all time intervals (Figure 2).

Table 2 shows the comparison of the time of the first rescue analgesic demand in the postoperative period. It was significantly early in Group T with a mean value of 5.69 ± 0.87 hr compared to Group Q with a mean value of 11.23 ± 2.22 hr.

**Table 2:** Comparison between the groups for the time of the first analgesia used Q = quadratus lumborum, T = transversus abdominis plane ^a^Two-sample proportion test *Highly significant

Groups	Time of first analgesic used (mean ± SD), hr
Group Q	11.23 ± 2.22
Group T	5.69 ± 0.87
P value^a^	0.0001*

The total number of analgesic demands required in the postoperative period up to 24 hr was compared between the two groups. It was found that in Group Q, 29 (82.85%) patients required analgesic two times in 24 hr and rest 6 (17.14%) patients required analgesic three times in 24 hr, whereas in Group T, 30 (85.71%) patients required analgesic three times and rest 5 (14.28%) patients required four times in 24 hr. Thus, it was significantly more in group T as verified based on the two-sample proportion test (Table 3).

**Table 3:** Comparison between the groups for postoperative demands for rescue analgesia Q = quadratus lumborum, T = transversus abdominis plane ^a^Two-sample proportion test *Highly significant

Total number of analgesic demands	Group Q	Group T	P value^a^
Demand 1 (diclofenac 75 mg)	0	0	-
Demand 2 (diclofenac 75 mg + diclofenac 75 mg)	29	0	0.0001*
Demand 3 (diclofenac 75 mg + diclofenac 75 mg + tramadol 100 mg)	6	30	0.0001*
Demand 4 (diclofenac 75 mg + diclofenac 75 mg + tramadol 100 mg + tramadol 100 mg)	0	5 (14.28)	0.027

**Figure 3:**
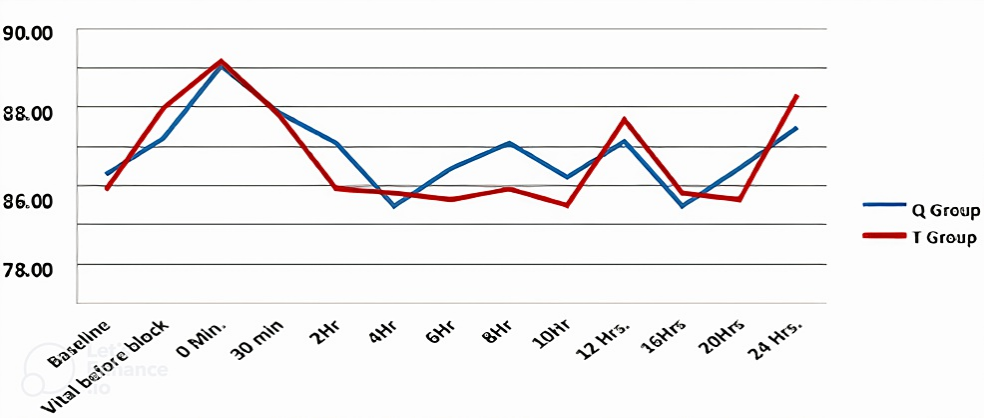
Comparison of heart rate (beats/min) between the groups across time periods Q = quadratus lumborum, T = transversus abdominis plane

The heart rate (beats/minute) was compared between the groups using the two-sample t-test across the time periods. It was found that there was no significant (P > 0.05) difference in HRs between the groups in all the time periods (Figure 3).

**Figure 4:**
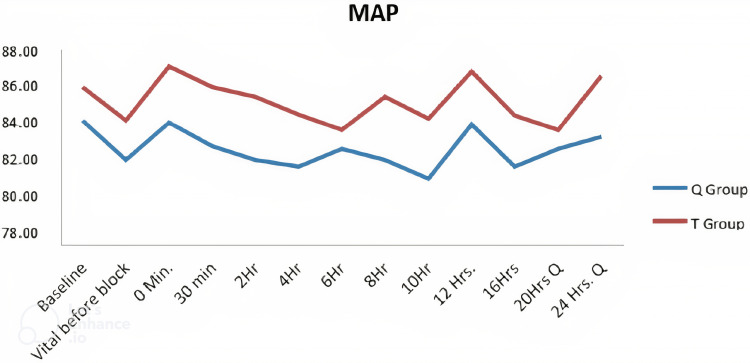
Comparison of MAP between the groups (mm Hg) Q = quadratus lumborum, T = transversus abdominis plane, MAP = mean arterial pressure

Similarly, MAP between the groups across the time periods was compared using the two-sample t-test. There was no significant (P > 0.05) difference in MAP between the groups in all time periods (Figure 4).

Table 4 shows the comparison of patient satisfaction between the groups. There was a highly significant (P < 0.001) difference in patient satisfaction between the groups; patient satisfaction was more in Group Q compared to Group T.

**Table 4:** Comparison of patient satisfaction between the groups SD = standard deviation, Q = quadratus lumborum, T = transversus abdominis plane ^a^Two-sample t-test *Highly significant

Groups	Patient satisfaction (mean ± SD)
Group Q	5.8 ± 0.41
Group T	4.74 ± 0.44
P value^a^	0.0001*

## Discussion

Postoperative pain after abdominal surgeries is intense. Adequate postoperative analgesia reduces the postoperative stress response, postoperative morbidity, and improves the surgical outcome. Abdominal truncal nerve blocks like TAP block and QL block are described for postoperative analgesia. A number of studies have been done to prove their analgesic efficacy. This study was conducted to compare the QL block (with the combined posterior and anterior approach) with TAP block for postoperative analgesia following open TAH under spinal anaesthesia. NRS scores for pain were observed at 0 min, and 2, 4, 6, 8, 10, 12, 16, 20 and 24 hr after the block. Time of the first demand of analgesics in the postoperative period or duration of analgesia, total analgesic requirement in 24 hr, patient satisfaction score and complications were also observed. 

In our study, the NRS score was higher in Group T at all time intervals and significantly higher at the fourth and sixth hour (P < 0.05) than Group Q. This was similar to the study done by Naaz et al., who compared the ultrasound-guided QL block and TAP block in patients undergoing TAH under general anaesthesia [11]. They found that the QL block group had a significantly better visual analogue scale (VAS) score from 6 till 24 hr postoperatively. A study by Jadon et al. showed similar results [12]. They found that the VAS score was lower in the QL block group at 2, 4, 6, 8, 12, 18, and 24 hr than the control group. Huang et al. compared the transmuscular QL block and oblique subcostal TAP block for analgesia after hysterectomy [13]. They found that NRS scores for visceral pain intensity were significantly lower in the QL group than TAP group. Liu et al. did a meta-analysis of eight randomized controlled trials involving 564 patients on QL block versus TAP block for postoperative analgesia in patients undergoing abdominal surgeries and got similar results [14]. The meta-analysis showed a higher pain score in the TAP group at 2, 4, 6, 12 and 24 hr. Verma et al. compared the QL block with TAP block for post-caesarean analgesia and found that the VAS score at rest and also with movement was less in the QL group than the TAP group [15]. Yousef compared the QL block and TAP block in patients undergoing TAH under general anaesthesia and observed that VAS scores were lower in the QL group than the TAP group [16].

In our study, the demand for the first rescue analgesia was significantly earlier in the TAP group than the QL group (P < 0.001). This was similar to the study by Naaz et al. [11]. The duration of analgesia was significantly more in the QL group (8.04 hr) than in the TAP group (5.59 hr). In the study by Jadon et al., the mean time to the first analgesic request was 7.8 hr in Group Q (block) and 3.2 hr in Group C (control) [12]. Huang et al. found that the time for the first morphine request was significantly later in the transmuscular QL group than the oblique TAP group [13]. Verma et al. in their study found that the time for the rescue analgesic was later in the QL group than the TAP group [15]. In the study by Yousef, the duration of postoperative analgesia in the TAP group was 8.33 ± 4 hr and 15.1 ± 2.12 hr in the QL group [16].

In our study, the total number of demands for rescue analgesia was significantly more in Group T than Group Q. This was similar to the study done by Naaz et al. who concluded that the cumulative analgesic consumption score was significantly lower in Group Q (mean = 0.74; 95% CI, 0.50, 0.98) compared to Group T (mean = 1.48; 95% CI, 1.26, 1.70) [11]. The mean dose of fentanyl utilized in 24 hr was considerably lower in Group Q compared to Group C in the study by Jadon et al. [12]. According to Huang et al., in the transmuscular QL group, total morphine consumption was considerably lower than the oblique subcostal TAP group (17.2 vs. 26.1 mg) [13]. Similar findings were obtained by Verma et al. in their study [15]. When compared to the TAP Group, the need for analgesics was much lower for more than 72 hr in Group QL. The number of patients requesting analgesia was substantially higher in the TAP group than in the QL group in the study by Yousef [16].

In our study, patient satisfaction was significantly higher in Group Q than Group T (P < 0.001). This was similar to the study by Öksüz et al., who found that the patient satisfaction score was higher in the QL block group [17].

In the present study, none of the participants experienced any serious postoperative side effects, such as bradycardia, hypertension, respiratory depression, nausea, vomiting, itching, or any drug-related reactions, that necessitated intervention. This was similar to the study done by Liu et al. [14]. They found no statistically significant difference between the two groups with regard to the incidence of postoperative nausea and vomiting (PONV). Similarly, in the studies conducted by Naaz et al. and Yousef, no significant side effects were observed due to QL and TAP blocks [11,16]. Deng et al. found that the incidence of dizziness in the QL group was lower than in the TAP group [18].

Limitations

There were also some limitations present in our study. Since this was an ultrasound-guided needle placement-based study, it was dependent upon the skills and expertise of the operator. We overcame this limitation by practicing blocks on about 15 patients before starting the study; a single investigator applied blocks to all patients. We did not compare the postoperative duration of the motor and sensory block that can affect the onset of pain in the postoperative period. We did not know the optimal dose of the local anesthetic (LA) for the QL block, and a higher dose or volume of LA might have improved and prolonged the analgesic effect. We excluded patients with a BMI > 30. Therefore, we do not know if these blocks have similar results in obese patients. Also, we did not assess the dynamic pain scores in this study. This was a single-center, small group study; multicentric studies are required with large sample sizes to clinically extrapolate the results.

## Conclusions

To conclude, the ultrasound-guided QL block given by combined type 2 and type 3 approach had better pain control at all time intervals, prolonged the duration of analgesia and reduced postoperative analgesic consumption after TAH when compared with the more established TAP block. These blocks were not associated with complications. We thus recommend to include the QL block as part of multimodal analgesia as its analgesic effect is more long-lasting than the TAP block and it can also avoid opioid-related side effects. Further trials are recommended to evaluate the ideal dose, volume and approach for the QL block.
